# Challenges in Mucosal HIV Vaccine Development: Lessons from Non-Human Primate Models

**DOI:** 10.3390/v6083129

**Published:** 2014-08-15

**Authors:** Iskra Tuero, Marjorie Robert-Guroff

**Affiliations:** Vaccine Branch, Center for Cancer Research, National Cancer Institute, National Institutes of Health, Bethesda, MD 20892, USA; E-Mail: iskra.tuero@nih.gov

**Keywords:** HIV vaccine, mucosal immunity, non-human primate

## Abstract

An efficacious HIV vaccine is urgently needed to curb the AIDS pandemic. The modest protection elicited in the phase III clinical vaccine trial in Thailand provided hope that this goal might be achieved. However, new approaches are necessary for further advances. As HIV is transmitted primarily across mucosal surfaces, development of immunity at these sites is critical, but few clinical vaccine trials have targeted these sites or assessed vaccine-elicited mucosal immune responses. Pre-clinical studies in non-human primate models have facilitated progress in mucosal vaccine development by evaluating candidate vaccine approaches, developing methodologies for collecting and assessing mucosal samples, and providing clues to immune correlates of protective immunity for further investigation. In this review we have focused on non-human primate studies which have provided important information for future design of vaccine strategies, targeting of mucosal inductive sites, and assessment of mucosal immunity. Knowledge gained in these studies will inform mucosal vaccine design and evaluation in human clinical trials.

## 1. Introduction

Several key advances in human immunodeficiency virus (HIV)/simian immunodeficiency virus (SIV) research have been made possible by the extensive use of non-human primates (NHP) as models for virus infection, vaccine evaluation and disease treatment. HIV is mainly acquired via either the genital or the gastrointestinal route. The study of SIV mucosal transmission in macaque models has illustrated how the virus rapidly infects local target cells, with subsequent dissemination to regional lymph nodes and then distal sites [1,2,3]. Disseminated infection is quickly accompanied by CD4^+^ T cell depletion in the gastrointestinal (GI) tract. T cells producing IL-17/IL-22, cytokines important for the maintenance of the mucosal barrier, are preferentially lost. The resultant immunological dysfunction and disruption of the structural barrier of the GI tract contributes to disease progression [4]. Therefore, an effective vaccine for preventing HIV infection most likely will require induction of immunity at mucosal sites. Most licensed vaccines are administered systemically by intramuscular or subcutaneous injection. However, mucosally delivered vaccines offer several advantages including non-invasive application, induction of both systemic and mucosal immune responses, and allowance of multiple booster immunizations [5]. Nevertheless, not only is assessment of induced mucosal immunity more difficult compared to systemic immunity, but also defining vaccine regimens to target key mucosal sites is complex. Rather than simply evaluating responses in blood, intensive and technically challenging protocols are needed to obtain mucosal tissue biopsies in order to phenotype and assay the functionality of mucosal T and B cells. Mucosal secretions are used to explore antibody responses and interactions with mucus components. However, different collection procedures and experimental approaches result in variability in sample quality and differences in results obtained. While a combination of mucosal and systemic vaccination might improve protection with regard to both blocking virus transmission and preventing systemic dissemination, the optimal route for delivery of mucosal vaccines is problematic, and may differ according to vaccine vehicle. In this regard, studies in NHP are invaluable in assessing mucosal immunization routes and comparing multiple vaccine platforms.

In this review, we draw heavily on NHP studies to provide an overview of different approaches used to assess cellular and humoral immune responses elicited by mucosal HIV/SIV vaccines, the contribution of different mucosal immunization routes to induction of protective immune responses and current progress in the development of mucosal vaccines against SIV/HIV. Together, the information provided identifies key issues, summarized in Table 1, that need to be addressed in order to develop an efficacious mucosal vaccine.

## 2. Evaluating Mucosal Immunity

Induction of mucosal immunity occurs in mucosa-associated lymphoid tissue (MALT). Therefore sampling of the mucosa is necessary for assessment of elicited immune responses and can be optimized in NHP. Most of the mucosal surfaces of the respiratory, GI, and urogenital tracts are lined by an epithelial barrier that provides non-specific and innate defenses including mucins and antimicrobial proteins. Epithelial cells detect dangerous foreign components through pattern-recognition receptors such as Toll-like receptors (TLRs), sending cytokine and chemokine signals to underlying mucosal cells including dendritic cells (DCs) and macrophages [6,7]. These trigger innate nonspecific defenses and together with intraepithelial lymphocytes and cells in the lamina propria, promote adaptive immune responses against pathogens such as HIV [8]. Use of the SIV-rhesus macaque model of the acquired immunodeficiency syndrome (AIDS) has provided evidence that cytotoxic T lymphocytes (CTL) in mucosal tissues play a crucial role in clearance or containment of SIV infection [9,10], and together with viral-specific antibodies might contribute to preventing establishment of virus reservoirs. The local production and secretion of HIV/SIV-specific secretory IgA and transudated IgG might also prevent mucosal cell infection and/or help control viral dissemination by virus entrapment and immune exclusion [11] or by other as yet unidentified mechanisms or functional activities such as neutralization and inhibition of transcytosis across the mucosal barrier [12,13,14,15,16,17].

**Table 1:** Key issues in SIV/HIV mucosal vaccine development.

- Differences among mucosal tissues in anatomy, physiology and immunology
- Compartmentalization of mucosal tissues regarding immune response induction
- Improvement of mucosal sample collection, processing and cryopreservation
- Optimization of immunization routes
- Characterization of trafficking and mucosal homing of T and B cells
- Identification of mucosal immune correlates of protection
- Development of mucosal cellular and humoral memory responses

With regard to mucosal barrier protection, mucus plays a key role but exhibits complexity in composition and viscosity as it mixes with other mucosal secretions, and in antibody isotype depending on mucosal site. Fahrbach *et al.* [18] have described how cervical mucus becomes less viscous as it travels toward the vagina and becomes cervical/vaginal mucus. The antibody isotype also changes in the female reproductive tract (FRT) with both IgG and IgA present in cervical mucus, but only IgG in cervical/vaginal mucus [18]. Both immunoglobulins can participate in binding and trapping pathogens and aid in their clearance. However, further knowledge of how these Ig isotypes are elicited and details of how their tight binding occurs will provide important information for vaccine design.

The complexity of mucosal secretions with regard to the presence of IgG and/or IgA requires care in ascribing functions to a particular isotype [19,20]. The described interference in effector function between different immunoglobulin isotypes in mucosal secretions [20] suggests that characterization of mucosal IgG/IgA following purification might provide more accurate information regarding *in vivo* function. A three-step affinity purification scheme has been described for IgA in human genital secretions [21] while purification of rhesus macaque mucosal IgA from fecal matter has used sequential protein G and jacalin/anti-monkey IgA columns in order to obtain large amounts of purified IgA ([22] Musich *et al.*, submitted). Human IgA purified from genital secretions and saliva of highly exposed seronegative individuals and from colostrum of HIV-seropositive women [23,24] as well as purified fecal IgA from vaccinated and/or SIV-infected rhesus macaques were shown to mediate transcytosis inhibition. The latter also possessed neutralizing activity and mediated antibody-dependent cellular phagocytosis (ADCP) [22].

Longitudinal monitoring of mucosal secretions can be routinely carried out in pre-clinical vaccine studies in NHP models and provides critical information regarding the ability of different vaccine regimens to elicit humoral mucosal responses, thus informing and facilitating vaccine design. Evaluation and quantitation of viral-specific antibody responses in mucosal secretions is more difficult than in peripheral blood due to a number of factors. The amount of total antibody in secretions is variable; thus the amount of specific antibody must always be normalized to the amount of total IgG or IgA present. A number of methods are used in collecting secretions, introducing additional variables. Vaginal and rectal secretions are often obtained from both humans and NHP using Weck-Cel sponges [21,25,26,27,28,29,30] or cotton swabs [14,15] placed in transport media containing protease inhibitors [17,21,27,30] and bovine serum albumin (BSA). The sponges and swabs can be frozen after collection for later analysis. Another strategy is the lavage method which involves washing the mucosal surface with a salt solution and then collecting the liquid for analysis [17,21,26,31,32]. Cervical fluids can be also collected using a small brush inserted into cervix, which is rotated and then placed in transport medium [21,27]. However, these latter approaches often greatly dilute the secretions and require immediate processing to remove epithelial cells and debris, which is not always feasible during clinical trials conducted in developing countries [21]. A procedure in which secretions are not diluted involves collection of cervical secretions with a mucus aspirating device [18] or a self-sampling method using a flexible menstrual cup frequently used for feminine hygiene [33]. In a comparative study using Weck-Cel sponges, lavage and brushing, better IgA and IgG recoveries from people were obtained using Weck-Cel sponges. The addition of EDTA, antibiotics and protease inhibitors to mucosal secretions improved the quality of collected mucosal IgA [21]. An alternative experimental approach to evaluate mucosal antibody is the use of explant tissue. Mucosal biopsies from human and macaque small intestine have been cultured to evaluate the ability of long lived plasma cells to secrete antibodies [34,35]. The duodenal explants mimic *in vivo* conditions including tissue architecture and survival factors produced by stromal cells, thus supporting long-lasting cell survival. Thomas *et al.* [36] used a similar strategy by culturing macaque rectal explants to monitor SIV Env-specific IgA. The method has the advantage that antibody can be collected over several days of culture, increasing the amount of mucosal antibody available for additional functional assays. Antibody secretion by rectal and duodenal explants of macaques was found to be similar. Thus, rectal biopsies are suitable for monitoring induction of mucosal antibody and facilitate studies, since rectal tissue is more accessible than duodenal tissue which requires invasive endoscopy or necropsy for collection.

Mucosal immunoglobulins are generally quantified by ELISA. For example, SIV Env-specific IgG and IgA have been assessed in mucosal secretions [37] and quantified using a set of calibrated standards [38]. As mentioned above, due to the variability in immunoglobulin content of secretions, in part attributed to different collection methods, the amount of specific Ig must then be normalized to the total amount of either IgG or IgA in the sample for accurate comparison among all samples. Often the amount of specific Ig in secretions is limited or below the limit of detection. Recently introduced microsphere-based methods are becoming popular for improving quantification of analytes in serum and mucosal secretions. Thus, by multiplex (suspension array-based) immunoassay, IgG antibodies to SIV_mac239_ Env, Gag, Pol and Nef were detected in sera and rectal secretions negative by ELISA in infected animals with evident viremia [29]. In addition to low Ig levels, additional factors can complicate analysis of antibody responses in mucosal fluids. In NHP, blood contamination is often a problem, making a determination of the origin of the Ig measured problematic. One solution is simply to quantify secretory IgA using an anti-secretory component antibody in a standard ELISA. This method results in lower antibody titers than one using anti-monkey IgA detection, but reliably detects secretory IgA. Using such a method, SIV Env-specific sIgA in rectal secretions was correlated with delayed SIV acquisition in rhesus macaques following an intrarectal SIV challenge [16]. Other issues include high concentrations of interfering proteins and glycans, and variations in female genital fluids according to the phase of menstrual cycle and hormone levels in both humans and NHP when monitoring cervical/vaginal secretions. [25,39,40,41]. Such factors should be considered when selecting the time of sampling for vaccine evaluation. Saliva is also a complex secretion, containing in addition to immunoglobulins, many endogenous factors which contribute to control of HIV infection, including mucins, cystatins, defensins, secretory leukocyte protease inhibitor, and lactoferrin [42]. However, these don’t generally complicate evaluation of antibodies. The saliva of SIV infected macaques was shown to exhibit increased development of SIV-specific antibodies over time, correlated with increased ability of the saliva to inhibit SIV infection [42]. Although not applied yet to the SIV macaque model for mucosal vaccine evaluation, analysis of human cervical secretions for immunoregulatory cytokines has been performed by multiplex assay, requiring only small volumes of sample [32,43,44] and providing additional information regarding innate and adaptive immune responses associated with protective efficacy.

Memory B cells can also be evaluated in mucosal tissues, providing another option for understanding development and maintenance of vaccine protective immunity. A novel flow cytometry method using direct staining of Env-specific memory B cells without initial cell enrichment has been developed [45], allowing quantification of this cell population in mucosal tissues as well as peripheral blood and bone marrow. Identification of plasma cells and plasma blasts in mucosal tissue by flow cytometry techniques is now also possible [46], and complements ELISPOT methods.

As outlined above, mucosal antibodies have been correlated with protection against SIV/SHIV infection in rhesus macaques [14,15,16,17]. Further, antibodies in general are believed important for vaccine-elicited protection in view of the modest efficacy seen in the Phase III clinical vaccine trial in Thailand, RV144, in which protection was correlated with antibodies in the V2 region of the HIV envelope, and with non-neutralizing antibody-dependent cellular cytotoxic activity (ADCC) [47,48]. However, T cell immunity may also contribute to enhanced protection [49,50]. In the SIV macaque model the critical role played by SIV specific CD4^+^/IL-2^+^ T cells in colorectal tissue, and by CD8^+^/IFNγ ^+^ T cells in vaginal tissue, in protecting against rectal and vaginal SIV challenges, respectively, both in mediating resistance to SIV infection and in long-term viremia control, has been documented [10,38,51]. The potent protection elicited by vaccination of rhesus macaques with rhesus cytomegalovirus vectors (RhCMV) carrying SIV genes in which approximately 50% of vaccinated animals control and/or clear pathogenic SIV to undetectable levels following mucosal challenge [52,53], is attributed to effector memory T cells present not only in blood, but in mucosal tissues.

As with evaluating mucosal humoral immunity, assessing mucosal cellular immune responses mediated by cells recovered from tissues is similarly complex. In the macaque model, monitoring of mucosal T cell responses over the course of a vaccine regimen was for some time accomplished by substituting bronchoalveolar lavage (BAL) cells for intestinal biopsies, as the lung is a T cell effector memory site, and was described as a “window” providing a representative view of T cells at an effector site [54]. Recently, however, with improved assay procedures for small numbers of cells, rectal pinch biopsies have become the norm, and are relatively easily obtained for monitoring immunity in the gastrointestinal tract. When sampling the FRT, however, both the timing with regard to the menstrual cycle and the biopsy site(s) must be carefully considered. The FRT can be divided into upper and lower components. The upper FRT, lined with single-layered columnar epithelium, includes the endocervix, uterine endometrium and fallopian tubes, while the lower FRT, lined by multilayered stratified squamous epithelium, includes the vagina and extocervix [55]. In addition to this difference in surface layers, hormones impact each site differently, modulating immune functions such as CTL activity and secretion of Igs, cytokines, and other biologically active molecules in the upper FRT throughout the menstrual cycle, with little impact on such processes in the lower FRT [55]. These differences are understandable if one considers the necessity of balancing protection against pathogens *versus* providing a period of tolerability and immune suppression to allow implantation and pregnancy. In fact the days following ovulation have been described as a “window of vulnerability” with regard to HIV infection, when immune suppression creates a more favorable environment for viral infection [56,57]. Consequently, the timing and site of cervical/vaginal biopsies will impact readouts regarding mucosal immunity. Sampling of cells of the upper FRT has been routinely performed using a cytobrush inserted into the female reproductive tract. However, only small numbers of cells are recovered, sufficient for phenotyping, but not functional assays [5,26]. To obtain higher cell yields, biopsies are needed. A recent study compared cells recovered using cervicovaginal lavages (CVL), endocervical cytobrushes, and ectocervical biopsies [58]. The CVL samples provided the lowest number of viable leukocytes, while two cervical cytobrushes provided similar numbers of cells as one biopsy. However, predominant cells in the cytobrush samples were macrophages, while the biopsies contained mostly T cells. Use of explant tissue has also permitted investigation of how HIV traverses the female FRT. Viral transmission is not limited to columnar epithelium of the upper FRT. Rather, virions can also penetrate squamous epithelium of the lower FRT in macaques [59].

In view of the small numbers of cells available for evaluation, the development of mucosal immunity and the specificity and functionality of mucosal T cells is generally monitored by flow cytometry using antigen-specific tetramers [28,60,61] and intracellular cytokine staining [16,28,62,63]. As shown by analysis of peripheral blood T cells in HIV progressors and non-progressors, the presence of polyfunctional T cells has been correlated with a better clinical outcome [64]. With the increasing power of multicolor flow cytometry, it became possible to evaluate even small numbers of mucosal cells for polyfunctional T cell responses which have been seen in both NHP rectal and vaginal tissue biopsies [10] and in the rectal mucosa of chronically-infected people [65]. Recently, a technique to enumerate antigen-specific responses by single T cells using a low number of cells (10^4^–10^5^) was reported. This approach called “microengraving”, combines on-chip imaging cytometry and capturing of secreted proteins [66]. For a better absolute quantification of lymphocytes in mucosal tissue samples the use of fluorescent bead-based quantification may provide more accuracy [67].

## 3. Mucosal Vaccination Routes

Among several advantages, preclinical vaccine studies in NHP allow optimization of mucosal vaccine delivery routes in order to achieve desired adaptive immune responses. MALT includes multiple mucosal immune inductive sites such as nasal- or oropharyngeal-associated lymphoid tissue (NALT) and gut-associated lymphoid tissue (GALT). These compartments are separated anatomically but are functionally connected. Induction at one mucosal site leads to an effector response at a distal mucosal site. Communication between inductive and effector sites is mediated by homing mechanisms on induced T and B cells. Mucosal immunity also involves interplay between innate and adaptive immune systems through a complex interaction of cellular and signaling networks [68,69,70]. Systemic immunization strategies may induce mucosal immunity, however it is not clear whether the induced immune response is as protective as that following mucosal immunization [5,71,72]. The combination of systemic and mucosal vaccination routes might potentially improve T cell and antibody responses both at the site of virus entry and after systemic dissemination [73].

### 3.1. Intranasal (IN) Vaccination

The nasal epithelium contains ciliated epithelial cells, mucous goblet cells and specialized nonciliated cells similar to M cells at other mucosal sites. The main immune cells at the nasal inductive sites are similar to those found in GALT [74]. The nasal immunization route is attractive due to easy, needle-free administration (as sprays or drops), the dense vascularization in a small area that allows use of low amounts of vaccine, and the fact that it is effective at inducing systemic and mucosal immunity in the gastric mucosa and genital tract [25,71,74,75,76,77]. However, a major obstacle regarding IN vaccination is the potential for side effects in the central nervous system and nasal epithelium [72]. Nasal vaccines therefore require stringent safety testing. Over the past few years, a variety of vaccine approaches have been tested by the IN route, including both live vectors, and non-replicating vaccines.

Among live attenuated vaccines, several have been administered solely by the IN route with no heterologous booster immunizations and induced mucosal immunity. For example, a live, attenuated NYVAC/SIV recombinant vaccine encoding *gag*, *pol* and *env* successfully induced Gag-specific CD8^+^ T-cell responses in macaques at both vaginal and rectal sites [78]. Similarly, nasal administration of a non-pathogenic *nef*-deleted SHIV to macaques elicited HIV Env-specific IgG and IgA antibodies in vaginal, rectal, and oral secretions [79]. Moreover, following intravaginal (Ivag) challenge with SHIV_89.6P_, three of four immunized macaques exhibited no detectable virus. Using live attenuated poliovirus as a delivery vector, IN immunization of cynomologus monkeys with poliovirus expressing SIV Gag, Pol, Env, Nef and Tat proteins induced both rectal and vaginal SIV-specific IgG and IgA [80]. Of seven vaccinated monkeys subsequently challenged vaginally with SIV_mac251_, 2 exhibited no detectable virus and 2 had significantly decreased viral loads.

Other vaccine strategies have combined IN with other mucosal immunizations, either simultaneously or sequentially. We have used replication-competent adenovirus recombinants based in an Ad5 host range mutant vector (Ad5hr) extensively in several pre-clinical vaccine studies. Following mucosal priming with live Ad5hr-recombinants and intramuscular (IM) boosting with envelope components, significant protection against SIV and SHIV mucosal challenges has been achieved in rhesus macaques [81,82]. Further studies to explore the basis for the vaccine-induced mucosal protection will be discussed in section 3. It is worth noting that live vectors in general elicit mucosal immune responses at multiple sites, regardless of their route of immunization, presumably due to their broad biodistribution. This is the case with attenuated NYVAC whether administered mucosally or intramuscularly [78], and certainly with replication-competent Ad. The latter vector, administered either intranasally/intratracheally, sublingually, vaginally, or rectally elicited comparable Env-specific secretory IgA responses in vaginal, rectal, and nasal secretions and in saliva, and similarly comparable cellular responses at mucosal sites, with the exception of the vagina where responses were more compartmentalized [16]. This breadth of immune response was attributed to persistent Ad replication targeting macrophages and antigen presenting cells in mucosal tissues [83].

Non-replicating vectors and other vaccine components have also been administered nasally, and have successfully elicited mucosal responses and varying degrees of protection against SIV and SHIV challenges. Nasal administration of plasmid DNA encoding HIV/SIV genes, with or without DNA encoding cytokines such as IL-2, IL-12, and IL-15, followed by heterologous boosting with modified vaccinia Ankara (MVA) encoding viral genes, has been reported in a series of studies. The DNA/MVA regimen elicited low level rectal antibodies as well as cellular responses in rectal tissue. However, the regimen including IL-2 resulted in the best control of CD4 T cell loss and disease progression following rectal SHIV_89.6P_ challenge [37]. IN *versus* IM administration of DNA/MVA regimens were compared in rhesus macaques in two separate studies, one using SHIV-DNA adjuvanted with IL-12 [84] and the other SIV-DNA adjuvanted with IL-2 and IL-15 [38]. In both cases nasal vaccination resulted in better preservation of CD4^+^ T cells in the gut following rectal challenge with SHIV and SIV, respectively. Similarly, in female rhesus macaques, a DNA/MVA regimen incorporating multiple cytokines elicited SIV specific CD8^+^ T cell responses in blood and mucosal tissue following nasal immunization with strong control of viremia observed following intravaginal SIV challenge [51].

Aside from the use of vectored vaccines for uptake and expression of vaccine immunogens, viral particles and proteins have been administered directly by the nasal route. The use of nanospheres as a potential delivery vehicle for vaccine antigens was explored using inactivated SHIV-KU-2 captured in polystyrene nanospheres [85]. Nasal immunization induced vaginal HIV-Env specific IgA and IgG antibodies and partial protection against an Ivag SHIV-KU-1 challenge. An alternative immunization approach used oligomeric HIV_SF162_ gp140ΔV2 Env-protein administered intranasally with LTK63 adjuvant and compared this with the same protein and MF59 adjuvant administered intramuscularly and with sequential IN/IM or IM/IN administrations of the same proteins [86]. The sequential IN/IM regimen elicited the highest IgG responses in vaginal and nasal washes and in saliva, and also the highest IgA responses in vaginal washes and serum. Surprisingly, following vaginal challenge with homologous SHIV_SF162P4_, the regimens that incorporated IM immunizations provided the greatest protective efficacy, showing no detectable plasma viremia. The protection was associated with serum neutralizing antibodies.

### 3.2. Oral Vaccination

The oral mucosa is a stratified squamous epithelium supported by the underlying lamina propria [87]. Within the epithelium, the predominant cells are APC and T cells, and together with macrophages, fibroblasts, and mast cells produce a range of cytokines providing protection against pathogens. The salivary glands, where plasma cells produce IgA, are surrounded by the lymphatic draining system and represent inductive and effector sites for immune responses [87]. Oral vaccination has the capability to induce immune responses mainly in the gut and mammary and salivary glands. However, potential disadvantages of this route include dilution of the administered antigen by saliva and loss of stability or degradation of the antigen by the acid pH of the stomach. In addition, some antigens administered orally may induce immune tolerance in the gastrointestinal tract [71,72]. The use of enteric-coated capsules designed to resist stomach acid and dissolve in the neutral pH of the intestine provides a solution that facilitates the appropriate uptake of antigen in the intestine [88]. Such capsules have been evaluated in the macaque model, where two sequential oral administrations of replicating Ad-recombinant vaccine were compared with an IN followed by oral administration. The IN/oral regimen was superior to oral/oral immunization in inducing systemic and mucosal Env-specific antibody responses, but both immunizations elicited similar SIV-specific cellular responses in blood and cells at a representative mucosal effector site, the lung, evaluated using cells in BAL [89]. Control of viremia following an intrarectal (IR) SIV_mac251_ challenge was seen in both immunization groups, although the IN/oral regimen resulted in significantly better control of acute viremia. This was shown to be correlated with higher serum binding antibodies that mediated ADCC activity [14]. The IN/oral group also exhibited stronger transcytosis inhibition by antibodies in rectal secretions [15].

An alternate procedure to avoid effects of stomach acid involves administering vaccines orally following neutralization of stomach acid with sodium bicarbonate. This type of oral administration has been applied in a number of macaque studies using oral Ad-recombinant priming in combination with IN immunization, and has elicited potent viremia control following both IR SIV_mac251_ and intravenous (IV) SHIV_89.6P_ challenges [81,90,91]. Non-vectored vaccines have also been orally administered in this fashion. SIVp55gag particles plus cholera toxin as an adjuvant given orally to macaques induced specific IgG and IgA systemically and in the gastrointestinal but not genital tract [92]. However, oral administration without buffering of stomach acid has also been used successfully in some cases. A replicating modified-Tiantan vaccinia (MVTT) virus-based vaccine administrated orally in PBS in combination with IN immunization induced robust Gag- and Pol-specific CD8^+^ T cell responses in peripheral blood [93], although mucosal immune responses were not evaluated.

The oral cavity includes the sublingual and buccal regions in the front and the pharynx and tonsils in the back. Thus “oral” vaccines may be administered to one of these sites rather than being delivered to the stomach. For example, a DNA/MVA strategy administered the vaccines to the cheek of rhesus macaques [94], successfully inducing resistance to SIV_mac251_ infection and longer survival [10]. The sublingual mucosa contains a heavy network of dendritic cells, and this route of immunization has been shown to elicit both systemic and mucosal immune responses against protein antigens coupled to cholera toxin [95] as well as protection against influenza challenge [96] in mice. With regard to HIV antigens, other murine studies have shown induction of viral-specific IgA in the genital mucosal following gp41 Env and reverse transcriptase-cholera toxin B administration [97], and both systemic and mucosal CTL responses induced by replication-defective Ad5-HIVgag vaccine [98]. We have evaluated sublingual (SL) priming with replication-competent Ad-recombinant vaccines. The vaccine vector was broadly distributed to mucosal sites including BAL and rectal tissue where it persisted in macrophages and antigen presenting cells, and elicited systemic and cellular immune responses comparable to those induced following administration to other mucosal routes [83]. Similarly, protective efficacy compared to IN/intratracheal (IT), IR and Ivag administration was not enhanced [16].

Vaccine administration to the oropharynx/tonsils can elicit mucosal immune responses at distal sites such as the genital and gastrointestinal tract [71]. Stahl-Hennig *et al.*, using vaccine regimens containing non-replicating Ad-SIV recombinants and/or single cycle immunodeficiency virus administered via an atraumatic spray onto the tonsils of rhesus macaques elicited strong peripheral SIV-specific IFN-γ-positive T-cells [99]. In a similar approach, a SHIV/MVA construct given to macaques intramuscularly, intradermally or to the palatine tonsils induced both peripheral Env-specific antibody and CD8^+^ T cells [100]. A study in macaques initially primed with DNA vaccines compared MVA boosting by an IM/intradermal route *versus* IM/intradermal administration followed by an oral spray onto the palatine tonsils [101]. The group that received the tonsillar immunization exhibited a dominant T cell proliferative response and better maintenance of CD4^+^ T cells post-SHIV_89.6P_ IR challenge. A similar study in which DNA priming was followed by either IM/oral spray or oral spray/IM administration of a recombinant Ad boost showed that giving the mucosal immunization first resulted in better immunogenicity, although both groups of macaques displayed similar protective efficacy following an oral SIV_mac239_ challenge [102]. Using a tonsillar mode of attenuated SIVΔnef vaccination, protection against a SIV_mac251_ tonsillar challenge was associated with increased dendritic cells and γδ T cells, suggesting innate immune responses might contribute to blocking of virus transmission [103]. When AT-2- inactivated SIV_mac239_ plus CpG as an immunostimulatory oligonucleotide was administrated via the palatine/lingual tonsils, no peripheral T and B cell responses were elicited but mucosal IgA was detected in rectal secretions. Following rectal SIV_mac239_ challenge, the vaccinated animals had a lower frequency of infection and somewhat lower acute viremia compared to controls, providing additional evidence that immunization by the oral route can benefit vaccine protective efficacy [104].

### 3.3. Intratracheal (IT) Vaccination

To target inductive sites in the upper respiratory tract, IN immunization is generally sufficient. However, enhanced mucosal immunity can sometimes be induced by targeting the bronchoalveolar lymphoid tissue, where the most prominent feature is the presence of large B cell follicles producing IgA at the bronchial bifurcation [105,106]. Marx *et al.*, for example, used inactivated SIV formulated in microspheres for IT immunization of rhesus macaques [107], and demonstrated induction of viral-specific antibody in vaginal secretions. One of three macaques immunized by this route was protected from an SIV vaginal challenge. We have routinely used IT administration of replication-competent Ad-recombinants in rhesus macaques, generally as a booster immunization following initial IN or IN/oral priming. The route provides a better “take” in the lungs, and leads to enhanced immunity in the face of anti-vector immunity elicited by the initial immunizations. These sequential routes of immunization were used in priming macaques with Ad5hr-HIV_89.6P_ Env followed by boosting with envelope protein and elicited neutralizing antibodies correlated with sterilizing protection in 3 of 4 vaccinated macaques [82]. A similar vaccine approach elicited multiple systemic and mucosal antibody activities and contributed to the control of viremia following SHIV_89.6P_ challenge of rhesus macaques [15]. On the other hand, although a single IT Ad-SIV recombinant boost and two envelope protein boosts following two sequential DNA immunizations induced good immune responses, the combination regimen did not elicit protection against a rectal SIV_mac251_ challenge [108].

### 3.4. Gastrointestinal (GI) Tract Vaccination

The colon and rectum make up the lower gastrointestinal tract and thus comprise part of the GALT. The rectum is one of the major mucosal HIV transmission routes. The rectal mucosa both in NHP and in humans contains many activated macrophages, cytotoxic T cells and plasma cells in the lamina propria, representing potential targets for SIV/HIV infection [8]. Immunization by the IR route in humans has been investigated for its ability to elicit local antibody responses in the colorectal mucosa, in order to provide protection against sexually transmitted diseases [25]. In fact, IR immunization might be a suitable alternative to oral immunization for inducing an immune response in the gut, while avoiding immunogen degradation by gastrointestinal enzymes. Although, rectal vaccination may not be attractive or acceptable in certain cultures, some studies have shown that it can induce both local and systemic responses [77]. A report exploring IR immunization in NHP using a synthetic multiepitope SIV/HIV peptide vaccine together with *E. coli* heat-labile enterotoxin (LT) adjuvant induced cellular immune responses at mucosal sites including the small intestine, and protected macaques against SIV/HIV infection, clearing the virus to undetectable levels in the intestine, the major viral reservoir, as well as in peripheral blood [109]. In a later study, Wang *et al.* [110] investigated the effects of IR vaccination with a DNA vaccine producing SHIV particles followed by boosting with the same DNA or with MVA encoding SIV *gag/pol* and HIV *env*. SIV- or HIV-specific IgA was elicited in rectal secretions, but only inconsistently and of short duration. Peripheral SHIV-specific T cell responses and CD4^+^ T cell preservation correlated with control of long term viremia after IR challenge with SHIV_89.6P_. In a similar set of experiments, Belyakov *et al.* [111] studied IR immunization with adjuvanted peptides (HIV Env, Tat and SIV Gag) and NYVAC encoding HIV *env* and SIV *gag*/*pol*, alone or in combination. The peptide/NYVAC combination elicited high peripheral CD8^+^ T cell responses and both CD8^+^ T cell tetramer binding in the colonic mucosa and CTL activity in mesenteric lymph nodes. High-levels of vaccine-induced mucosal CTLs correlated with delayed systemic dissemination of virus following an IR SHIV-Ku2 challenge. IR priming with replicating-Ad5hr recombinants expressing SIV Env/Rev and Gag elicited central memory (CM) and effector memory (EM) T cells systemically and at mucosal sites, while Env-specific antibody was detected in blood and secretory IgA was detected in several mucosal tissues [16]. In this study, Env-specific rectal IgA was correlated with delayed SIV acquisition. Finally, intracolorectal immunization in NHP to target mucosal inductive sites has been explored using TLR agonists plus IL-15-adjuvanted viral-specific SIV peptides in combination with MVA vectors expressing SIV genes [60,112]. Innate immune and CD8^+^ T cells responses were induced systemically and some immunized animals developed gp120 binding antibodies. Further, significant viral-specific mucosal CD8^+^ T cell tetramer responses were elicited. The surprising finding of these studies, however, was the role of adjuvant alone in conferring partial protection against an SIV_mac251_ IR challenge. Further exploration of this effect is warranted.

### 3.5. Intravaginal (Ivag) Vaccination

In the FRT, the ectocervix and vagina consist of stratified squamous epithelia with a greater proportion of Langerhans cells (LC) in the extocervix than in the vagina. In contrast, the endocervix and upper reproductive tract consist of simple columnar epithelium, lacking LC. The single layer of cells may facilitate HIV-1 entry [59]. There are only limited studies exploring vaginal immunization, due in part to the strong effect of the menstrual cycle that induces alterations in the epithelial layers of the FRT, thus introducing greater complexity into immunization scheduling. Additionally, immune responses elicited are usually weak because of the lack of secondary lymphoid tissue as an inductive mucosal site. Alternative routes for inducing optimal immune responses in the vaginal mucosa include IN/ oral and systemic immunization [74,77]. Nevertheless, several studies have achieved vaginal immune responses. Vaginal application of trimeric HIV-1_CN54_ Clade C gp140 followed by IM immunization elicited HIV-specific IgG/IgA systemically and vaginally but only in animals that had seroconverted. Moreover, the administration of an IM immunization following Ivag priming boosted both systemic antibody and antibody in cervical and vaginal secretions [113]. In an effort to develop a vaccine that blocks vaginal transmission of HIV, the vaginal mucosa was targeted with human papillomavirus pseudovirion vaccines delivering SIV *gag* DNA [28]. The Ivag immunization of macaques elicited both local and systemic SIV-specific T cells and antibody responses. However, following SIV_mac251_ Ivag challenge, protection against infection was not achieved. Whether this novel immunization approach merits further investigation, including use of immunogens in addition to SIV Gag, is problematic, in view of the strong CD4 T cells responses elicited which might provide additional targets for viral infection. Ivag immunization has also been evaluated using replicating Ad5hr recombinants encoding SIV genes. Although both systemic and vaginal CD4^+^ CM and EM cellular immune responses were elicited, the induced immunity was compartmentalized, showing limited extension to other mucosal sites [16], although SIV-specific secretory IgA (sIgA) responses were elicited at all mucosal sites evaluated. Vaccine strategies using non-replicating helper-dependent Ad vectors (HD-Ad) in which all Ad genes have been deleted to avoid the host immune response against Ad proteins, have also been administered intravaginally. Macaques previously infected with Ad5 were immunized intravaginally with a series of heterologous HD-Ad recombinants expressing HIV-1 Env. CD4^+^ T cell responses were more highly induced in the colon than in blood by this immunization route [62]. Neither vaginal cellular responses nor antibodies in secretions were assessed. Finally, Ivag immunization with a DNA/MVA SIV vaccine regimen stimulated a mucosal T cell response and efficiently induced sIgA in vaginal secretions [10]. However, oral and nasal administration of the same vaccine elicited better protective efficacy.

## 4. Immune Correlates of Vaccine-Induced Mucosal Protection in NHP

While mucosal surfaces represent sites of vulnerability for HIV and SIV infection, they also represent the first line of defense of the immune system for protection against viral transmission, replication, and disease progression. Understanding how mucosal immune responses are optimally induced and the mechanisms of vaccine-elicited protection can help the rational design of mucosal vaccines [1,114]. Mucosal vaccines can trigger both humoral and cell-mediated immune protection not only at mucosal sites but also systemically. After vaccination, mucosal barriers against infection are reinforced through the induction of antigen-specific sIgA antibody which prevents pathogens adhering to or infecting epithelial cells and breaching the mucosal barrier [115]. In addition, specific effector T cells are distributed to mucosal sites to reinforce this barrier by cytokine production and/or cytotoxic activities [73]. To facilitate mucosal vaccine development, defining the immunological correlates of mucosal protection is critical. Some evidence has emerged from studies on HIV-exposed sero-negative subjects revealing the involvement of multiple factors such as mucosal HIV-specific antibody, low-level T-cell activation, and expression of IFN-γ and IP-10 that correlate with resistance to sexually transmitted HIV infection [43,116]. However, studies in NHP models have been invaluable in identifying immune responses associated with mucosal protection.

Viral-specific CD8^+^ T cells have emerged as a key player in mucosal protection. The most effective vaccine to date, live-attenuated SIV, elicits strong protection against a superinfecting pathogenic SIV administered by either the IV or mucosal routes [117,118] but requires a significant period of time for immune responses to mature before protection is achieved. The mode of protection against vaginal challenge has been attributed to CTL activity, as neutralizing antibodies were not elicited [118]. However, this does not preclude an effect of other innate or adaptive immune responses. In a study examining tetramer^+^ T cells in mucosal tissue, no increase in the number of such viral-specific cells was observed between 5 and 20 weeks after SIVΔnef vaccination, casting doubt as to whether CD8^+^ T cells were responsible for protection against SIV_mac251_ vaginal challenge following “maturation” of the immune response [119]. The authors discuss the possibility that the quality of the CD8^+^ T cells may have changed over time, or alternatively that other immune responses developed that contributed to the observed protection by decreasing the viral burden, allowing the CTLs to control early founder populations. Similar observations were made by others using an attenuated SHIV_89.6_ vaccine which mediated protection against Ivag SIV_mac239_ challenge. Depletion of CD8^+^ T cells from the immunized macaques prior to challenge eliminated protection [120]. However, while the CD8^+^ T cells were required for protection, other effects of attenuated SHIV_89.6_ infection likely made a significant contribution, including decreasing circulating plasmacytoid dendritic cells, suppressing T cell activation, decreasing mRNA levels of proinflammatory mediators, and increasing mRNA levels of immunoregulatory molecules [121].

Perhaps the clearest evidence for the role of vaccine-induced cellular immunity in preventing mucosal as well as systemic infection comes from studies using RhCMV vectors encoding SIV genes. These vaccines have been shown to elicit persistent, high-frequency SIV-specific effector memory T cell responses leading to potent control and clearance of SIV_mac239_ infection in approximately 50% of macaques challenged intravenously, intrarectally, or intravaginally [52,53]. The CD8^+^ T cells elicited by RhCMV are unique in recognizing diverse, promiscuous epitopes including some restricted by MHC class II molecules [122], and thus may not be representative of other vaccine-induced CD8^+^ T cells. However, other vaccine approaches have also linked cellular immunity to protection against mucosal challenge. Immunization with DNA and MVA encoding SIV genes preserved colorectal CD4^+^ CM T cells and protected macaques from progression to AIDS following an IR SIV_mac251_ challenge [38] while in female macaques resistance to Ivag SIV_mac251_ infection and persistent suppression of SIV viremia was associated with vaginal CD8^+^ T cells and mucosal IgA responses [10]. In addition, a number of studies have correlated systemic cellular immunity to protection from mucosal viral challenge. Vaccine regimens studied include replicating Ad5hr-SIV recombinant mucosal priming and IM Env protein boosting, leading to no viremia or potent control of viremia in 39% of immunized macaques following SIV_mac251_ IR challenge. Control of viremia at the set point of infection was significantly associated with CD8^+^ T cell activity [81]. The protection was durable, and continued to show a correlation with cellular immunity as verified by CD8^+^ T cell depletion [63]. The novel replicating modified-Tiantan vaccinia virus-based SIV vaccine evaluated by IN/oral priming and an IM boost with a non-replicating Ad5-SIV recombinant showed reduced peak viremia, control of chronic viremia, and protection from disease progression after IR SIV_mac239_ challenge. The protective effects were correlated with a SIV Gag/Pol-specific CD8^+^ T cells producing IFN-γ and TNF-α [93]. A variety of vaccine regimens combining non-replicating Ad recombinants with DNA or poxvirus vectors have also shown a correlation of cellular immunity in peripheral blood with viremia control post-mucosal challenge [123,124,125]. Similarly, a DNA/listeria monocytogenes oral vaccine regimen elicited cellular immunity that led to control of viremia following IR SIV challenge [126]. DNA vaccines with or without an inactivated virus particle boost have also conferred viremia control following IR SIV_mac251_ challenges, significantly correlated with systemic cellular immunity [127,128]. Whether such viremia control results solely from control of systemic viral replication following viral dissemination from initial founder populations of infected cells, or whether control is initiated earlier via immune cells in mucosal tissues will have to be determined by further studies focused directly on mucosal tissues.

The humoral arm of the immune system has also been clearly linked to vaccine-induced mucosal protection. Neutralizing antibodies are a current key focus of vaccine research and early on were shown to be able to prevent mucosal transmission of SHIV isolates. Baba *et al.* [12] demonstrated that IV administration of neutralizing monoclonal antibodies to neonatal macaques led to their subsequent protection against a SHIV-vpu^+^ oral challenge. Likewise, Mascola *et al.* [13] showed that IV administration of neutralizing monoclonal antibodies to adult rhesus macaques provided protection against a subsequent Ivag SHIV_89.6PD_ challenge. These proof-of-concept experiments were followed by studies that demonstrated the ability of vaccine regimens to elicit potent neutralizing antibodies significantly correlated with protection against mucosal SHIV challenges. Significant protection against a homologous Ivag SHIV_SF162P4_ challenge was obtained using an oligomeric HIV-1gp140_SF162ΔV2_ vaccine administered intramuscularly, intranasally, or by both routes. While Env-specific IgG and IgA were elicited at mucosal sites, high serum IgG antibody titers with neutralizing activity against the homologous virus appeared to correlate with protection [86]. Using the same immunogen, HIV-1gp140_SF162ΔV2_, expressed in alphavirus replicon particles (VRP) together with SIVgag and pol, IM, IN, and IR administration of the VRP were compared followed by boosting with Env protein. The best protection was afforded by the intramuscularly primed vaccine regimen, and following IR challenge with SHIV_SF162P4_, was correlated with serum neutralizing antibody against the homologous virus [129]. Mucosal priming with replicating Ad5hr-HIV_89.6P_*env* followed by boosting with HIV_SF162_ gp140 protein also led to sterilizing immunity in 3 of 4 vaccinated macaques following an IR HIV_SF162P4_ challenge correlated with neutralizing antibody [82].

Other properties and activities of vaccine-elicited antibodies in addition to neutralization have been significantly correlated with protective efficacy. Antibody avidity has been identified as a correlate of mucosal protection in the VRP study above [129] as well as following vaccination with DNA/MVA regimens [130,131], an ALVAC/Env regimen modeling the RV144 clinical trial [132], and with replicating Ad5hr-recombinant/Env boost approaches [16]. Among non-neutralizing antibody activities associated with vaccine-induced protection against mucosal SIV and SHIV challenges, ADCC has been extensively studied. Gomez-Roman *et al.* [133] initially reported a significant correlation of ADCC activity elicited by a replicating Ad5hr-SIV recombinant priming/Env protein boosting regimen with reduced viremia in rhesus macaques following IR challenge with SIV_mac251_. This finding has been reproduced in other studies using the same replicating Ad-based vaccine approach [14,15,16,134]. Importantly, it has also emerged as a correlate of complete mucosal immune protection following vaccination with the highly effective live-attenuated SIV vaccine [135]. A related activity, antibody-dependent cell mediated viral inhibition (ADCVI) [136] has also been associated with vaccine-induced protection from mucosal challenge. The activity includes ADCC but also can exert an effect by secretion of cytokines or by opsonization. It has been associated with protection of neonatal macaques from oral SIV challenge [137]. Furthermore, the activity often appears in conjunction with ADCC, and is similarly correlated with protection from mucosal challenge [14,15,16,134].

Antibodies both in serum and mucosal secretions mediate inhibition of transcytosis, a mechanism by which HIV and SIV can traverse epithelial cell barriers [138]. Both IgG and IgA antibodies present in secretory fluids of HIV-positive individuals have been shown able to inhibit this transcytosis [23,24], illustrating another mechanism by which non-neutralizing antibodies might contribute to vaccine-elicited protection against mucosal infection. We have shown that vaccine elicited antibodies in plasma and mucosal secretions are correlated with decreased acute [14] and chronic [15] SIV and SHIV viremia respectively. Similarly, apparent sterilizing immunity in rhesus macaques against Ivag challenge with SHIV_SF162P3_ following immunization with gp41 subunit virosomes was correlated with gp41-specific IgA in vaginal fluids able to inhibit transcytosis [17], while at the same time, neutralizing antibodies were absent in sera of the macaques. This suggests that antibodies in mucosal secretions can contribute to protective efficacy by mechanisms other than simply binding virions [18], thus blocking their transmission across mucosal barriers. In this regard, we have shown that SIV Env-specific secretory IgA in rectal secretions was significantly correlated with delayed SIV acquisition [16]. The mechanism of this protection remains to be elucidated. A method developed to purify large quantities of mucosal IgA from fecal samples may allow characterization of vaccine-induced IgA in order to determine functional activities and investigate protective mechanisms ([22], Musich *et al.*, submitted).

The contribution of the humoral arm of the immune system to mucosal protection has also been shown by direct study of B cells in vaccinated and challenged NHP. Study of memory B cells by ELISPOT analysis elicited by a replicating Ad-recombinant vaccine regimen has shown correlations with functional antibody activities including ADCC, and transcytosis inhibition prior to and after SIV mucosal challenge [139]. Post-challenge memory B cells were significantly correlated with reduced chronic viremia. Further, a recent study has correlated the presence of plasma cells in rectal tissue of female rhesus macaques with delayed acquisition following IR SIV challenge ([140], Tuero *et al.*, in preparation). Further investigation of humoral components in mucosal tissues will undoubtedly lead to clarification of immune protective mechanisms and vaccine regimens for their optimal induction.

Overall, immune correlates analysis has shown that both the cellular and humoral arms of the immune system contribute to mucosal protection. Often both components are seen to play a role, making identification of a single mechanism problematic. A spectrum of immune responses is likely necessary for protection against both HIV and SIV acquisition as well as control of viremia and disease progression resulting from transmission events that are not prevented. Studies in NHP can lead to optimized vaccine regimens able to elicit the necessary multiplicity of immune responses.

## 5. Clinical Trials

Worldwide, more than 35 million people live with HIV, and only 9.7 million have access to antiretroviral treatment in low and middle-income countries [141]. Therefore, it is imperative to develop an effective HIV vaccine able to induce broadly protective immune responses at the viral portal of entry. The vast majority of human clinical trials has used IM delivery of vaccine components and has focused on elicitation of systemic responses including neutralizing antibodies, CTL responses, or a mixture of humoral and cellular responses [49]. Results of the RV144 multicenter, randomized, double-blind, placebo-controlled phase 3 efficacy trail provided new hope in the field of HIV vaccine development, achieving modest protection of 31% [142]. The protective efficacy was associated with V1/V2 binding antibodies, low level Env-specific serum IgA, and ADCC activity [47,48]. The field has now begun to consider mucosal vaccine approaches in addition to systemic regimens, which may lead to greater efficacy. However, as indicated on the Clinical Trials Website [143], not many such trials have been conducted. A few of the more recent trials are mentioned below.

Mucosal vaccination has been explored in women using different vaccine systems. A phase I double blind randomized controlled trial was conducted using HIV-1 CN54 clade C recombinant trimeric envelope (gp140) designed to mimic the native spike of the envelope protein on the virion surface [27]. The immunogen was applied vaginally in Carbopol gel. No adverse effects were induced in the volunteers. Although the vaccine had induced systemic and mucosal humoral immune responses in rabbits [144,145] and was able to effectively prime and boost mucosal immune responses in cynomolgus monkeys [113], the regimen was poorly immunogenic in women. Cervico-vaginal IgG and IgA responses were either not elicited or were sporadic. Moreover, cervical gp140 specific T cell responses were not detected. The different responses in rabbits compared to humans might relate to differences in anatomy and physiology of the genital tract between the species. However, in view of the more positive outcome in the NHP model, systemic boosting of vaccinated women might have elicited both systemic and mucosal antibody responses.

Broad neutralizing antibodies, including 2F5 and 4E10, interact with the membrane-proximal external region (MPER) on gp41 adjacent to the viral membrane. Thus gp41 is an attractive immunogen for HIV/SIV vaccine design. In a randomized phase I trial, healthy women were vaccinated with virosomes containing a lipidated HIV-1 gp41 P1 peptide by sequential IM and IN administrations. The vaccine was safe and well-tolerated. P1-specific IgG antibodies were induced systemically and in vaginal and rectal secretions, and showed transcytosis inhibition activity, but no P1-specific T cell responses were elicited in blood. HIV-specific transcytosis inhibition by vaginal secretions correlated with the presence of P1-specific IgG antibodies while IgA was poorly induced [30].

Based largely on the numerous studies in NHP demonstrating protective efficacy elicited by mucosal immunization with replicating Ad-recombinants, a Phase I study of safety and immunogenicity of replication-competent Ad4-HIV vaccine vectors encoding *gag* and *env* genes in healthy volunteers is now recruiting volunteers. The study will initially evaluate oral administration, and subsequently delivery to the upper respiratory tract. The replicating Ad4 platform was initially clinically evaluated in a phase I trial of an oral Ad4-flu vaccine, and was shown to be safe and effective in priming a subsequent boost with inactivated flu vaccine [146]. A prototype Ad4-HIV*env* vaccine was shown to elicit both humoral and cellular immune responses in rabbit and mouse models, including neutralizing activity [147].

With the advent of more clinical trials aimed at assessing mucosal immunization strategies, the field has recognized a lack of reliable methods for evaluating mucosal immunity. Efforts are underway to improve immunological analysis of mucosal secretions. The clinical trial “Study of immunity at the genital mucosa of HIV-1 infected and healthy women (MUCOVAC) (identifier NCT01715103)” will explore the utility and tolerance of cytobrush and cervicovaginal washing in women infected or not infected with HIV-1 [143]. However, additional methods to assess immunity at mucosal sites will need to be developed.

## 6. Concluding Remarks

Mucosal transmission of HIV is mediated by exposure to virus and/or infected cells within mucosal secretions. Blocking virus entry at mucosal sites and subsequent dissemination to distal tissues is the goal of a preventive mucosal HIV vaccine. SIV/HIV mucosal vaccine research in NHP, by defining key antibody and cellular immune responses important for protection following mucosal challenge, facilitates the rational development of HIV vaccines. To evaluate vaccine efficacy, identification of phenotypic signatures of mucosal resident T and B cells, including trafficking and functional properties, is necessary and can be pursued vigorously in NHP models. Analysis of mucosal secretions also permits identification of interactions between cellular and molecular components including mucus proteins, cytokines, chemokines and antibodies. NHP use also allows assessment of sampling methods for optimal results.

The challenges in evaluating mucosal immune responses include numerous hurdles regarding mucosal sample collection and the lack of reliable and sensitive techniques for evaluating humoral and cellular responses at mucosal sites. The ability to select optimal sampling sites and define methods and optimal storage and transportation conditions will lead to improved sample quality, facilitating identification of desirable immune responses for a mucosal vaccine. Developing reliable methods suitable for mucosal secretions and tissues in macaque models should translate to the human system, providing solid and reproducible results that permit robust evaluation of candidate mucosal HIV vaccines.

Few reports have shown either induction of innate immune responses following vaccination or correlation of such responses with protection. However, as the innate immune system is the first line of defense against viruses, bacteria and parasites, it may be important to elucidate how dendritic cells influence the adaptive immune response at systemic and mucosal sites, and to determine how vaccines and adjuvants activate the innate immune system. Other components of the innate system, such as NK, NKT, and γδ T cells may also have relevant biological roles in vaccine-elicited immune responses and control of HIV infection. It is beyond the scope of this review to cover these aspects of innate immunity, however, recent reviews are available [148,149]. Continued research of both innate and adaptive mucosal immunity with regard to protection against mucosal transmission of HIV will contribute to rational vaccine design. Further, if robust protection is not elicited by mucosal vaccination alone, the combination of mucosal and systemic immunizations can be assessed in the NHP model for greater protective efficacy.

The use of NHP as models of SIV and SHIV infection has led to many important advances in understanding the biology of HIV infection in humans. Moreover, pre-clinical testing of vaccine regimens in NHP has greatly facilitated development of systemic vaccine approaches. In on-going studies, these models will be invaluable for development of mucosal vaccines. Evaluation of candidate mucosal vaccines in NHP should accelerate the goal of attaining an effective HIV mucosal vaccine.
